# Comparison of vaginal breech deliveries with and without magnetic resonance imaging in primigravidas: a retrospective cohort analysis and literature review

**DOI:** 10.1007/s00404-024-07915-2

**Published:** 2025-03-07

**Authors:** G. Cinari, A. Edner, A. Rody, K. Kraft

**Affiliations:** 1 University of Lübeck, Lübeck, Germany; 2 Department of Obstetrics and Gynecology, University of Lübeck Hospital, Ratzeburger Allee 160, 23538 Lübeck, Germany

**Keywords:** Pelvimetry, Vaginal breech delivery, Sonography criteria for vaginal breech, Breech outcomes

## Abstract

**Purpose:**

Pelvimetry is often recommended in primiparous patients before offering vaginal breech delivery. Later studies show a reduction in perinatal mortality in women undergoing pelvimetry while earlier studies show the opposite. Magnetic resonance imaging (MRI), considered a new technology in 1990, has become the more expensive method for pelvimetry with lower-radiation, believed to prevent unnecessary cesarean sections and “falsely attempted vaginal deliveries”.

**Methods:**

This retrospective cohort study (November 2019–February 2024) involved 160 primigravidas with breech presentation. The deliveries were attended by a team of experienced obstetricians (defined as attending at least 20 vaginal breech deliveries per year). Our cohort without MRI was compared with four study cohorts with MRI that were also used in a 2022 systematic review assessing delivery outcomes.

**Results:**

Neonatal outcomes, cesarean section rate and vaginal delivery rate were compared. Umbilical artery pH was significantly lower in two study cohorts (Hoffmann et al. 2016 7.18 vaginal vs. 7.24 caesarean section (*p* < 0.001), our cohort 7.19 vaginal vs. 7.27 cesarean section (*p* < 0.001)). The vaginal delivery rate without MRI (our cohort) was 65.6%. In studies with prior MRI as a selection criterion, the rate was between 65.4% and 67.5% (Hoffmann, Van Loon, Klemt). 25.5% of our patients who had to be delivered by cesarean had non-reassuring fetal heart tones in the second stage of labor. Only 4.4% of the patients attempted delivery with epidural anesthesia.

**Conclusion:**

Pelvimetry has not been shown to predict neonatal outcome and there is still no consensus on the interpretation of MRI measurements. Many authors argue, as confirmed by our results, that the outcomes are not dependent on pelvimetry, but on the competence of the obstetric delivering team.

## What does this study add to the clinical work

**Table:** 

This study shows that pelvimetry with magnetic resonance tomography does not influence outcomes, does not prevent unnecessary cesarean sections, and does not prevent “falsely attempted vaginal deliveries”. The vaginal rate of breech delivery and outcomes were similar among primigravidas attempting delivery without magnetic resonance imaging pelvimetry, leading us to conclude that the clinical experience of the delivery team and sonographic criterion were more influential than pelvimetry on outcome.	

## Introduction

The rationale for magnetic resonance imaging (MRI) in women attempting vaginal breech delivery has been cited as a method to reduce adverse neonatal outcomes [1, 2]. While breech presentation estimates vary between 4 and 6%, it is often recommended that a pelvic assessment be performed before offering a vaginal delivery, especially among primiparous patients [3]. In contrast, vertex positions are considered as “normal” and the fetal head is considered as a marker for the pelvis.

The amount of vaginal breech deliveries and physicians performing them declined rapidly after the publication of the Term Breech Trial [4–7]. Estimates from the Netherlands showed a 30% increase in cesarean rate of breech babies within the first 2 months following publication [8].

Pelvimetry has been recommended over many years for patients attempting breech delivery [9, 10]. Although later studies have cited a reduction in perinatal mortality for women undergoing pelvimetry, earlier studies showed the opposite. Rovinsky reported a higher rate of perinatal morbidity and mortality among women with pelvimetry when compared to women without [11].

Magnetic Resonance Imaging (MRI) replaced classic x-ray pelvimetry in the 90’s. Although more expensive, the ionizing radiation exposure to mother and fetus was eliminated. It was speculated that MRI could prevent “unnecessary cesarean sections” and “wrongly accepted vaginal delivery” [1, 2]. The number of studies assessing outcome following MRI pelvimetry for women attempting vaginal breech delivery are extremely limited.

Although controversial, we believe outcomes are not affected by MRI or pelvimetry and that vaginal breech delivery can be attempted using sonographic criteria alone. We report the results of 160 primigravida women with breech presentation with intended vaginal delivery without pelvimetry or MRI, comparing our results to other published studies from 1997, 2016, and 2019 [2, 12, 13].

## Methods

This retrospective cohort study included 160 primigravidas with breech presentation who attempted vaginal delivery between November 2019 and February 2024. The study was reviewed by the Institutional Review Board (Number 2023-752 and 2024-297). Informed consent was obtained at the time of hospital admission. The data were analyzed retrospectively and anonymously. The deliveries were attended by a team of experienced obstetricians with experience in breech deliveries (defined as performing at least 20 vaginal breech deliveries yearly). The patients were screened, beginning in the 36th week of pregnancy. A portion of the cohort attempted external cephalic version. This was not a requirement for a trial of labor by breech presentation. After choosing a trial of labor, the patients were examined weekly, including fetal biometry, doppler examination, and cardiotocogram. Patients that had an indication for induction before their delivery date (preeclampsia, doppler abnormalities, oligohydramnios, induction desired (offered earliest at 39 ^0/7^ weeks) were induced with the same medications as patients over their due dates. The latest induction was started in the 40 ^3/7^ week of pregnancy. All inductions were performed with prostaglandin gel (Minprostin^®^E2 vaginal gel, Pfizer Pharma GmbH, Berlin). Sonographic measurements were obtained by a single sonographer. Biparietal diameter, anterior–posterior abdominal diameter, and transverse abdominal diameter were used in counseling and patient selection. Differences between biparietal diameter and abdominal diameter > 2 cm were considered as high risk for head entrapment and were not delivered vaginally. Patients with poorly controlled gestational diabetes, type 1 and type 2 diabetes, triple loop nuchal umbilical cord, class 3 obesity or higher, estimated fetal weight < 2000 or > 4000 g (sonography), severe growth restriction, and patients with general contraindication for vaginal delivery (including placenta praevia) were excluded. Parameters analyzed included the rate of deliveries (vaginal, secondary cesarean, emergency cesarean) with comparison of Apgar scores, umbilical artery pH, and birthweight. Statistical analysis was performed using SPSS (IBM^®^ Version 29). Independent *T* Test was used for means comparisons. A *p* value < 0.05 was used for statistical significance.

The studies used for literature review and comparison were variable. Hoffmann et al. was single center, retrospective with 240 patients over 8 years [12]. Van Loon was a randomized control trial over 3 years with multiple centers and 235 patients [2]. Klemt was single center cohort study over an eight-year period [13]. The majority of the studies performed MRI pelvimetry due to primiparity.

## Results

Demographic data are provided in Table 1, delivery characteristics in Table 2, neonatal outcomes in Table 3.

**Table 1:** Demographic data

*N* = 160 (primigravidas)	
Age (years)	32^a^ Range 22–40
Induction of Labor	76/160 (47.5%)
GA > 40 0/7	21/160 (13.1%)
Frank breech	100/160 (62.5%)
Complete or incomplete breech	38/160 (23.8%)
Not recorded	22/160 (13.8%)

^a^Median

Values are n/total (%)

**Table 2:** Delivery characteristics (*n* = 160 Primigravidas)

Vaginal delivery	105/160 (65.6%)
Epidural	7/160 (4.4%)
Cesarean section	55/160 (34.4%)
secondary	52/160 (32.5%)
emergency^a^	3/160 (1.9%)
Obstructed labor, first stage	12/52 (23.1%)
Obstructed labor, second stage	7/52 (13.5%)
Failed Induction attempt	9/52 (17.3%)
Non-reassuring fetal heart rate first stage	5/52 (9.6%)
Non-reassuring fetal heart rate second stage	14/55^b^ (25.5%)

^a^defined as immediate indication for delivery due to high risk for fetal asphyxia with general anesthesia

^b^includes 2/3 emergency cesarean sections due to bradycardia

**Table 3:** Neonatal outcome

Weight at delivery (grams)	3205 (1970–4250)
Female	93/160 (58.1)
Gestational age at delivery (weeks)	39 2/7 (33 4/7–41 3/7)
Apgar at 5 min	10
Umbilical artery pH	7.22 (6.98–7.54)
Base excess	− 7 ( − 18.9–3.2)

Values reported are median (Minimum – Maximum)

Comparison of our cohort to other study cohorts can be found in Table 4.

**Table 4:** Comparison of studies with and without MRI

Study	Study design	Number of patients	MRI criteria	Exclusion criteria	Secondary cesarean section	Emergency cesarean section	Vaginal delivery ^a^	Neonatal outcome
Hoffmann et al. 2016 [12]	Retrospective cohort, monocentric, primipara	240 (January 2006–August 2014, 8 years)	CV > 12 cm, performed 37.5 ± 1.6 gestational weeks	EFW < 2500 g or > 3800 g	78/240 (32.5%)	^b^	162/240 (67.5%)	No adverse events, pH 7.18 (vaginal) vs. 7.24 (cesarean), *p* < 0.001
Van Loon et al. 1997 [2]	Randomized control, multicenter	235 (January 1993–April 1996, 3 years)	CV > 11 cm, performed 38 weeks + 2 days (mean, range 36 weeks + 4 days – 42 weeks)	EFW > 4000 g	^b^	MRI 22/104 (21.2%) Control 41/113 (36.2%)	MRI 68/104 (65.4%) Control 58/113 (51.3%)	Lower Apgar in 6 women with pelvic abnormalities (control group, *p* < 0.01)
Lallemant et al. 2021 [20]	Observational, retrospective, comparative, bi-centric	559 (August 2013–August 2019, 6 years)	CT 36 -37 weeks, AP^c^ and SP^d^	EFW > 3800 g, Magnin’s Index < 23	AP^c^ 2/29 (7%) SP 76/341 (22%)	^b^	AP 27/29 (93.1%) SP 265/341 (77.7%)	No significant differences, no significant adverse events
Klemt et al. 2019 [13]	Cohort, monocentric, primipara	367 (January 2009–July 2017, 8 years)	CV > 12 cm, performed after 34 weeks + 0 days	EFW < 2500 g, GA > 41 ^0^^/7^ weeks	126/367 (34.3%)		241/367 (65.7%)	No significant differences
Cinari 2024	Retrospective, cohort, primipara	160 (November 2019–February 2024, 4 years)	^b^	EFW < 2000 g > 4000 g	52/160 (32.5%)	3/160 (1.9%)	105/160 (65.6%)	pH vaginal 7.19 vs 7.27 cesarean, p < 0.001

^a^planned vaginal delivery

^b^not described or not applicable

^c^asymmetric pelvis (AP), defined as ≥ 1 cm between the right and left oblique diameters

^d^symmetric pelvis (SP), defined as 2 oblique diameter measurements with a difference < 1 cm

The median age at the time of delivery in our cohort was 32 years (Range 22–40). Median delivery weight was 3205 g. When comparing the vaginal delivery group to the cesarean group, the weights differed significantly (3140 g vs 3490 g, *p* = 0.002). Apgar scores (median 5- and 10 Minute Apgar 10) and gestational age at delivery were consistent in both the vaginal and cesarean group (39 2/7 vs 39 3/7 weeks). Induction rates were higher in the cesarean group (65.4% vs 38.1%). The overall epidural rate was low (4.4%), with 85.7% of the epidurals in the cesarean group. The groups differed significantly in umbilical artery pH (7.19 in the vaginal group vs 7.27 in the cesarean group, *p* < 0.001). The vaginal delivery rate without MRI was 65.6%. The literature review using prior MRI as a selection criterion, showed a rate between 65.4% and 67.5%.

## Discussion

Multiple studies report improved neonatal outcomes for neonates with breech presentation born per cesarean compared to vaginal delivery [4, 8]. Rietberg calculated the need for 175 cesareans in order to prevent one neonatal death [8]. Vlemmix reported 338 cesareans to prevent one perinatal death in their population-based study [5]. Authors have warned about potential consequences of the increasing rate of cesareans, including fetal loss due to uterine rupture and maternal death resulting from multiple cesarean sections. [5, 8, 14–16]. We reported differences in umbilical artery pH 7.19 vaginal vs 7.29 cesarean. These results were consistent with Hoffman et al., both results statistically significant (*p* < 0.001), allowing us to conclude that our outcomes are comparable without the use of MRI [12].

Due to the absence of adequate training breech presentation has become one of the main diagnoses for women with primary cesarean. Studies in the United States have shown high rates among primigravidas (89.6–95.7%, Robson Criteria 6) [17]. Even among multiparas the cesarean rates were high (Robson Criteria 7, 90.2–93.9%). Trends in other countries are similar.

The use of MRI for patient selection remains controversial. Hoffmann et al. reported interspinous diameter (ISD) as the only MRI parameter that showed an association with outcomes and recommended its use over conjugate vera (CV) in the selection process. They additionally noted that pelvic flexibility during delivery should be considered in selection but made no recommendations on methodology [12].

Von Bismarck et al. used diameter transversalis (DT) and CV for selection for vaginal delivery. The recommended cesarean group had a smaller CV when compared to vaginal delivery, elective cesarean, and unplanned cesarean (*p* < 0.0001). Elective cesarean and recommended cesarean varied in DT (*p* = 0.039). There were no significant differences in pelvic inlet measurements between the groups (vaginal delivery, unplanned cesarean, elective cesarean) [18].

Walkinshaw commented on the MRI study conducted by van Loon, noting that it was unclear how MRI could increase the rate of successful vaginal delivery, arguing for clinical assessment over MRI [19]. Our results are consistent, showing that clinical assessment was more influential for outcome than MRI parameters and results.

Van Loon’s premier study included 35 women with abnormal pelvic measurements. The overall cesarean section rate was lower in the MRI group but the elective rate was higher (both not statistically significant). The authors reported a lower rate of emergency cesarean sections in the MRI group and significantly more complications when comparing the vaginal delivery group to the cesarean group (*p* = 0.01) [1]. Augmentation and induction of labor resulted in more emergency cesarean sections. While our induction rate was significantly higher in the cesarean group, we could not confirm the increase in emergency cesarean sections as a result. The authors reported lower Apgar scores among six women with “pelvic abnormalities.” [1] Lallemant et al. compared pelvic asymmetry in patients with CT pelvimetry and found no differences between asymmetric and symmetric pelvises [20].

Multiple studies have noted that pelvimetry cannot be used in the prognosis of neonatal outcome and there remains no consensus on the interpretation of the measurements. Many authors argue that the outcomes are not dependent on pelvimetry, but on the expertise of the teams treating the patients in the delivery room [19, 21]. Our patients were delivered by obstetricians with at least 20 vaginal deliveries per year. We too found that this was a determining factor in neonatal outcome.

It has been argued that delivering breech in an upright position results in fewer neonatal birth injuries, cesarean sections and shorter second stages of labor [22]. Michel et al. reported a wider pelvic diameter in squatting or upright positions, but smaller CV, contradicting the use of CV for patient selection [23]. CV is often used as criteria for allowing a trial of labor in patients with breech presentation [6, 7, 10, 13]. Our patients were not restricted during delivery, and we did not experience any differences in delivery rates or outcomes based on birthing position.

A 2022 systematic review including 18 studies concluded that there was no marker that could predict outcome. The review included patients with MRI during labor. Fourteen of 18 studies (77.8%) assessed fetuses in vertex presentation. Only four studies involving breech presentation were included (all cited in Table 2). In spite of the small number, the authors concluded that the studies may have excluded women with smaller CV without considering the fetal proportions, in turn eliminating women that may have successfully delivered vaginally. Additional critique was related to missing statements on head entrapment [24]. We did not experience any head entrapments among our cohort. Women with a higher risk of head entrapment were not offered vaginal delivery.

A more recent study argued that MRI pelvimetry can be helpful for the patient when she is deciding to attempt a breech delivery. Ebling et al. reported an increased willingness to attempt vaginally delivery following MRI, when the measurements were shared with the patients. This was unfortunately a small study with questionnaires for 32 women [25]. Tresch et al. attempted to assess the value of modern pelvimetry, assessing 551 patients with CT pelvimetry. They discovered that CV and ISD have not evolved since the mid twentieth century and argued (as we have) that there is no current consensus on pelvic measurements for women attempting breech delivery. The authors were critical to note that roughly 20% of their patient population would by current standards considered to have a “narrow pelvis”, arguing that the currents standards may be too restrictive for patient selection [26].

## Conclusion

There remains no consensus on MRI parameters for prediction of successful breech delivery in primiparas. In addition, there are no valid parameters for neonatal outcome prediction. Multiple studies have shown that clinical expertise is a better predictor of outcome.

Strengths of this study include the large amount of single center deliveries over a short amount of time, a stabile delivery team, and single sonographer criteria for patient selection. Weaknesses include the retrospective nature of the study. One could argue that the studies for literature review were limited and outdated but studies with primiparas attempting breech delivery are extremely limited. Although it can be argued that more studies are needed, multiple studies have already shown that clinical expertise is a better predictor than expensive diagnostics such as MRI. This study shows that omitting MRI for this patient population could result in a significant reduction in healthcare costs and resources without increasing perinatal morbidity or mortality.

## Abbreviations

CV: Conjugate vera
DT: Diameter transversalis
ISD: Interspinous diameter
MRI: Magnetic resonance imaging
